# Regulatory Functions of Hypoxia in Host–Parasite Interactions: A Focus on Enteric, Tissue, and Blood Protozoa

**DOI:** 10.3390/microorganisms11061598

**Published:** 2023-06-16

**Authors:** Emily DeMichele, Olivia Sosnowski, Andre G. Buret, Thibault Allain

**Affiliations:** 1 Department of Biological Sciences, University of Calgary, Calgary, AB T2N 1N4, Canada; emily.demichele1@ucalgary.ca (E.D.); olivia.sosnowski@ucalgary.ca (O.S.); aburet@ucalgary.ca (A.G.B.); 2 Inflammation Research Network, University of Calgary, Calgary, AB T2N 1N4, Canada; 3 Host-Parasite Interactions, University of Calgary, Calgary, AB T2N 1N4, Canada

**Keywords:** *Giardia duodenalis*, protozoa, hypoxia, HIF, *Entamoeba*, Leishmania, *Plasmodium*, *Cryptosporidium*, parasite

## Abstract

Body tissues are subjected to various oxygenic gradients and fluctuations and hence can become transiently hypoxic. Hypoxia-inducible factor (HIF) is the master transcriptional regulator of the cellular hypoxic response and is capable of modulating cellular metabolism, immune responses, epithelial barrier integrity, and local microbiota. Recent reports have characterized the hypoxic response to various infections. However, little is known about the role of HIF activation in the context of protozoan parasitic infections. Growing evidence suggests that tissue and blood protozoa can activate HIF and subsequent HIF target genes in the host, helping or hindering their pathogenicity. In the gut, enteric protozoa are adapted to steep longitudinal and radial oxygen gradients to complete their life cycle, yet the role of HIF during these protozoan infections remains unclear. This review focuses on the hypoxic response to protozoa and its role in the pathophysiology of parasitic infections. We also discuss how hypoxia modulates host immune responses in the context of protozoan infections.

## 1. Introduction

Normoxia describes a state in which a tissue is adequately oxygenized and able to carry out aerobic metabolism. This state is typically achieved with an external atmosphere consisting of ~21% oxygen, which translates to ~145 mmHg pO_2_ at sea level [1,2]. A coordinated network of oxygen gradients exist in the lungs, extending to all organ systems. In homeostasis, organs including the brain, lungs, and liver generally have an oxygen concentration ranging from 3.4% to 6.8% [3]. The gastrointestinal (GI) system exhibits one of the steepest oxygen gradients in the body, ranging from >33 mmHg in the proximal small intestine to 3 mmHg in the distal colon, subjecting the mucosa and local microbiota to fluctuating oxygen concentrations [4,5]. In vertebrates, intense exercise and insufficient environmental oxygen availability can cause hypoxia, a state that in turn engages anaerobic metabolism. Cell injury during hypoxia is generally reversible, in stark contrast with the irreversible damage incurred in anoxic conditions [6,7]. Hypoxia has been widely characterized in various cancers, organ pathologies, and metabolic diseases. Beyond the realm of cancer and chronic diseases, hypoxia plays an important role in infectious diseases, leading to changes in cellular metabolism that may contribute to or protect against pathophysiology. This article provides a state-of-the-art review on the role of tissue hypoxia and HIF activation in the context of parasitic infections, with a focus on tissue, blood, and enteric protozoa.

## 2. Hypoxia and Hypoxia-Inducible Factor

### Mechanisms of Tissue Hypoxia: HIF, Prolyl-Hydroxylases (PHDs), and Metabolism

Tissues can detect and respond to hypoxic conditions, wherein cells attempt to mitigate harm and promote survival during this undesirable state. Hypoxia-inducible factor (HIF) is one of the main players in the cellular detection of hypoxia. HIF is composed of two subunits: the constitutively expressed alpha subunit and nuclear beta subunit which dimerize to form the HIF complex [8]. The HIF complex needs to be readily available to rapidly modulate subsequent gene expression. There are three known alpha subunit isoforms (HIF-1α, HIF-2α, and HIF-3α), each of which dimerize with the HIF-1β, also known as the aryl hydrocarbon receptor nuclear translocator (ARNT) [9,10]. HIF-1α is generally expressed in all nucleated cells and contributes to many transcriptional regulatory processes in response to hypoxia. Although HIF-2α shares many similar target genes to HIF-1α, HIF-2α exhibits more restrictive expression [9,11]. A shift from predominantly acting HIF-1α to HIF-2α occurs during prolonged hypoxia [12,13]. HIF-3α, the most recently identified HIF-α, is primarily expressed in kidneys and lung epithelial cells, contributing to more specialized responses to hypoxia [14,15,16]. Under normoxic conditions, prolyl-hydroxylases (PHDs) and factor-inhibiting HIF (FIH) carry out their primary function, using oxygen as a substrate. PHDs specifically hydroxylate HIF-α isoforms on (one or two) conserved proline residue(s) in the oxygen-dependent degradation domain (ODDD), which allows HIF-α isoforms to be recognized and ubiquitinated by the von Hippel–Lindau protein (VHL), an E3 ubiquitin, and subsequently targeted for proteasomal degradation [9,10,17,18]. FIH-induced hydroxylation of HIF-α isoforms prevents the binding of p300/cAMP response element-binding protein (CREB) cofactors to the HIF complex [19,20,21]. Thus, under normoxic conditions, the dimerization of the HIF complex is inhibited as PHDs and FIH have sufficient oxygen substrates.

Under hypoxic conditions, HIF-α isoforms are stabilized and further translocate to the nucleus where they dimerize with HIF-1β, acting as a transcription factor for numerous genes. Dimerized and nuclear HIF will bind to hypoxia response elements (HREs) present in the promoter of target genes whilst bound to p300/CREB cofactors [19,20,21]. Families of genes that are regulated by HIF include genes involved in cellular metabolism, angiogenesis, proliferation, survival, combatting oxidative stress, redox homeostasis, epithelial barrier function maintenance, and erythropoiesis [9,10,11,22]. HRE consensus sequences (5′-(A/G)CGTG-3′) have been identified in the promoter of hundreds of genes, suggesting dramatic and widespread effects of HIF-1 stabilization [23]. However, other HRE sequences have been elucidated, including the HIF ancillary sequence (HAS) (5′-CAGGT-3′) [22]. The main targets of HIF are genes aimed at combatting cell oxidative stress and promoting angiogenesis. Importantly, HIF-1 is the immediate activator of proangiogenic genes such as vascular endothelial growth factor (*VEGF*) and erythropoietin (*EPO*), while HIF-2 is responsible for activating genes promoting vascular maturation, including *VEGF* receptors (*VEGFR1–3*), angiopoietin growth factors (*ANG1*, *ANG2*, *ANG3/4*), and their associated receptors (*TIE1/2*) [23,24,25,26]. The activation of hundreds of genes by HIF-1 is a complex process that may further rely on the activation of other transcription factors. For instance, heat shock transcription factor 1 (HSF1) can subsequently regulate HIF-1 signaling [27]. However, this transcription factor responds to heat shock, suggesting that activation of genes such as heat shock protein 70 (HSP70), a known HIF target gene, may also be alternatively regulated by stressors independent of oxygen.

Metabolic reprogramming is another biological target of HIF, helping cells sustain themselves in the absence of oxidative phosphorylation (OXPHOS). To do so, HIF-1 activates genes encoding the enzymes phosphoglycerate kinase-1 (*PGK1*), enolase (*ENOL*), aldolase (*ALDA*), lactate dehydrogenase-A (*LDHA*), and pyruvate dehydrogenase kinase (*PDK1*), effectively increasing glycolytic flux and inhibiting OXPHOS [28]. Other classes of HIF target genes include those promoting self-renewal (i.e., *ADN*, *EDN1*, *PGM*, *GPI*), proliferation (i.e., *CD73*, *ITF*, *MET*, *CTGF*), apoptosis (i.e., *BNIP3/3L*, *NRDG*, *NOXA*), and redox homeostasis (*GPX3*, *HMOX1*, *SOD2*) genes [22].

## 3. Protozoan Parasites and Hypoxia

Protozoa are known to engage in symbiotic or parasitic relations with their mammalian hosts. Due to specific tissue tropisms, many parasites exhibit a certain oxygenic affinity. Considering the steep, dual oxygenic gradients that exist throughout the gut, enteric parasites must be well adapted to the environment to establish an infection and proceed through their life cycle. As a result, numerous protozoans that colonize the GI tract are accustomed to low oxygen concentrations and may require microaerophilic conditions to survive and thrive. In this section we discuss the role of oxygen and hypoxia in the parasitism of enteric as well as tissue and blood protozoa.

### 3.1. Enteric Protozoan Parasites: Oxygen Metabolism and Host Hypoxic Response

#### 3.1.1. Hypoxia in the Gastrointestinal Tract

In the GI tract, hypoxia occurs upon low blood oxygen concentration or as a result of poor blood flow. Blood flow is important when it comes to gastrointestinal hypoxia as the amount of blood is highly dependent on meal consumption [1,29,30]. Several structural and biological factors can futher determine the oxygen levels in the GI tract, such as the region of the intestine, the mucosal layer, and the gut microbiota [1,4,9,29,30,31,32,33,34]. There are two oxygen gradients in the gut: (i) a longitudinal oxygen gradient, decreasing from the small intestine to the colon, and (ii) a radial gradient, with oxygen levels decreasing from the submucosa to the lumen [1,4,31]. The small intestinal radial gradient ranges from <10 mmHg in the lumen up to 59 mmHg, while the colonic radial gradient ranges from as low as 3 mmHg and 11 mmHg in the lumen (sigmoid and ascending colon, respectively) to as high as 42–71 mmHg in the muscularis externa [4,31]. Intestinal epithelial cells (IECs) are therefore reliant on HIF to promote cellular survival and adaptation when oxygen fluctuations subject them to a state of hypoxia [35]. Furthermore, inflammation in the GI tract leads to an influx of immune cells, resulting in a heightened hypoxic state due to depleted oxygen availability and altered cellular metabolism [36]. Colorectal cancer severity and metastasis have both been linked to increased tissue hypoxia. HIF activates numerous genes involved in tumor proliferation and angiogenesis [37,38,39,40]. For example, HIF-1α can also promote activation of ataxia-telangiectasia mutated kinase to protect colorectal cancer cells against apoptosis, an important mechanism by which tumors utilize hypoxia for enhanced survival [41]. Antibodies against HIF target gene products such as VEGF can further increase the duration of survival in patients when used in combination with other chemotherapeutic drugs [42].

IECs can also utilize the hypoxic response for protection against various system perturbations. For instance, *Clostridium difficile* toxins increase transcription and subsequent translation of HIF-1α in a dose-dependent manner, suggesting that IEC HIF-1α is protective against *C. difficile-*induced injury and inflammation [43]. Clinical symptoms of murine TNBS-colitis are exasperated when HIF-1α is decreased and, conversely, overexpression of HIF-1α can be protective [44]. Furthermore, hypoxia is implicated in various inflammatory conditions wherein cellular responses to hypoxia can promote the resolution of inflammation [36,45].

#### 3.1.2. *Entamoeba histolytica*

*Entamoeba histolytica*, the causative agent of amebic dysentery or amebiasis, is a leading cause of diarrheal illness in humans, with 50 million symptomatic infections per year [46]. After ingestion, cysts undergo excystation into trophozoites, which travel to the colon where they colonize and invade the epithelial tissue [46]. During this process, trophozoites traverse a steep gradient from the oxygen-poor colonic lumen to the oxygenated lamina propria [47]. *E. histolytica* has developed a complex machinery against nitric oxide (NO) and reactive oxygen species (ROS) released by neutrophils and macrophages to prevent macromolecular damage during the tissue invasion process, which subsequently maintains a strict state of intracellular hypoxia [48,49,50,51]. Oxygen is one of the main host stressors that influences *E. histolytica’s* survival and pathogenicity. *E. histolytica* can upregulate its antioxidant enzyme machinery, which includes iron superoxide dismutase, peroxiredoxin, and thioredoxin to evade host immune defenses [49,52,53,54].

**Figure 1:**
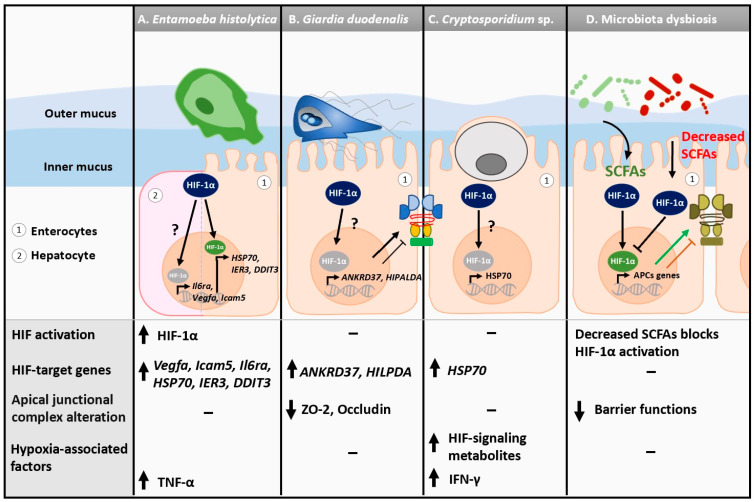
Alterations to mucosal HIF-1α expression and hypoxia-related signaling during enteric protozoan infections and microbiota dysbiosis. (**A**) Murine hepatocytes exhibit elevated levels of HIF-associated genes *Icam1* and *Il6ra* in response to *Entamoeba histolytica* infection. Human intestinal xenografts transplanted into SCID mice show elevated expression of several hypoxia-related genes, including *HSP70*, *IER3*, *DDIT3*, and *HIF-1α*, upon *E. histolytica* infection. (**B**) *Giardia duodenalis* (WB) is associated with increased expression of HIF target genes *ANKRD37*, *HILPDA, NOS2*, *GADD45A*, *HIG2, ITF*, *MIR210HG*, and *SLC2A3.* Expression of tight junctional proteins ZO-2 and Occludin are decreased upon *Giardia* infection in anaerobic conditions. (**C**) *Cryptosporidium muris* infection is associated with elevated fecal HIF-1-associated signaling metabolites. Hypoxia-induced heat shock protein 70 (*HSP70*) is elevated in response to *Cryptosporidium parvum.* (**D**) Microbiota dysbiosis can lead to decreased secretion of short-chain fatty acids (SCFAs), lessening HIF activation and barrier function. Although enteric parasites are associated with microbiota dysbiosis, no direct link between HIF signaling and microbial-secreted metabolites has been established in the context of enteric protozoan infections.

Alterations to HIF-1α expression and hypoxic-related signaling have been described during amebiasis (Figure 1A). *E. histolytica* is associated with hepatic hypoxic responses as HIF-1α and HIF-dependent genes *Vegfa*, *Icam1*, and *Il6ra* are upregulated in a murine model of *E. histolytica* infection [55] (Table 1). Interestingly, HIF-1α is required for adequate secretion of IL-6, indirectly modulating the anti-*E. histolytica* Th17 immune response [55]. Human intestinal xenografts transplanted into SCID mice exhibit upregulated expression of numerous genes associated with hypoxic responses upon *E. histolytica* infection, including those coding for HIF1α, metallothioneins (*MT1G*, *MT1H*, *MT1P*, *MT2A*, *MT3*), members of the c-Jun and c-Fos family *(JUN*, *JUNB*, *JUND*, *FOS)*, growth arrest and DNA damage inducible 3 (*DDIT3*), alpha crystallin (*CRYAB*), immediate early response 3 (*IER3*), and heat shock protein 70 (*HSP70*) [56] (Table 1). Furthermore, intestinal biopsies from humans infected with *E. histolytica* show elevated HIF activation, as well as enrichment for the response to hypoxia as a biological process [57]. Considering *E. histolytica* is an anaerobe, this finding suggests that the parasite may modulate its microenvironment to make the conditions more favorable, triggering an HIF-dependent cellular response.

#### 3.1.3. *Giardia Duodenalis*

The intestinal protozoan parasite *Giardia duodenalis* (syn. *G. intestinalis*, *G. lamblia*) causes giardiasis, one of the leading causes of diarrheal disease worldwide with over 200 million symptomatic cases annually in humans [73,74]. Clinical manifestations of giardiasis include acute presentations of diarrhea and abdominal pain, as well as post-infectious disorders such as post-infectious irritable bowel syndrome (PI-IBS), chronic fatigue, and failure to thrive in children [75]. After ingestion via the fecal–oral route, cysts undergo excystation and trophozoites colonize the upper small intestine by attaching to the surface of enterocytes [76,77]. *Giardia* is classified as a microaerophilic parasite, lacking mitochondria and enzymes for the electron transport chain (ETC) and tricarboxylic acid (TCA) cycle [9,78,79]. Instead, *Giardia* has a fermentative style of metabolism, utilizing glycolysis and substrate-level phosphorylation to generate energy while also possessing oxygen-scavenging enzymes such as flavodiiron proteins (FDPs), peroxiredoxin, flavohemoglobin, superoxide reductase, NADH peroxidase, and NADH oxidase to combat luminal ROS [80,81,82,83,84]. The ability to process oxygen also depends on *Giardia*’s life stage, wherein *Giardia* trophozoite uptake of oxygen is stimulated via exogenous glucose while cyst oxygen uptake is triggered by ethanol [85].

*G. duodenalis* has demonstrated the ability to activate a hypoxic-like response in cells (Figure 1B). Transcriptomic analysis on human duodenal organoid-derived monolayers infected with *G. duodenalis* strain WB clone 6 exhibit an enrichment of hypoxia response-related genes (i.e., Molecular Signatures Database) after 24 h of infection [86]. Similar observations have been made using Caco-2 epithelial cells, where HIF-induced genes including *NOS2*, *ANKRD37*, *GADD45A*, *ITF*, *MIR210HG,* and *SLC2A3* are also upregulated in *Giardia*-exposed cells, suggesting that IECs respond to increased oxidative stress upon infection [22,58,59,87] (Table 1). Furthermore, hypoxia-inducible gene 2 (*HIG2*), a peptide inhibitor of adipose triglyceride lipase encoded by a target gene of HIF-1 named *HILPDA* (Hypoxia-Inducible Lipid Droplet-Associated), is upregulated early on in infection, indicating that HIF activation may occur quickly upon cellular interaction with *Giardia* [58,88] (Table 1). However, little is known about the mechanisms and roles of HIF activation upon exposure to *Giardia*.

#### 3.1.4. *Cryptosporidium* spp.

*Cryptosporidium* species such as *Cryptosporidium hominis* and *Cryptosporidium parvum* are leading causes of diarrheal illness [89]. *Cryptosporidium* oocysts excyst upon ingestion into four sporozoites, which subsequently invade IECs and progress through their life cycle intracellularly [90]. As *Cryptosporidium* migrates from the gut lumen to the IECs, sporozoites are exposed to fluctuating but predominantly oxygen-poor environments [90]. *Cryptosporidium* can carry out both aerobic and anaerobic metabolic pathways, encoding a pyruvate:NADP+ oxidoreductase and an alternative oxidase, respectively [91,92]. This suggests that *Cryptosporidium* can not only tolerate but also make use of the variable oxygenic conditions of the gut [92]. Interestingly, the oocysts are present in higher abundances in water samples with lower dissolved oxygen, implying the oocyst may be more sensitive to oxygen than the invasive merozoites [93].

In a neonatal rat model of cryptosporidiosis, cardiomyocytes have hyperexpression of HIF-1α; however, this finding is more suggestive of a link between gastroenteritis and cardiovascular disease than a direct induction of hypoxia by *C. parvum* [60]. In the GI tract, the fecal HIF-1 signaling-associated metabolite oxoglutaric acid is increased in *C. muris*-infected mice, suggesting that mice can enact a hypoxic response upon *Cryptosporidium* infection [94] (Figure 1C). Finally, in HCT-08 colonic monolayers infected with *C. parvum*, heat shock protein 70 (*HSP70*), which possesses a hypoxia response element, is also upregulated 24 h post infection [61] (Table 1). The full biological significance of these observations requires further investigation.

#### 3.1.5. Hypoxia and Gut Epithelial Barrier Functions during Protozoan Infections

During states of inflammation and hypoxia, epithelial barrier function can be reduced as a result of the dysregulation of various tight junction proteins, including claudin-1 [95,96,97]. To combat this, the hypoxic response activates genes such as *ATG9A* and *CLDN1* to promote tight junction biogenesis and barrier integrity [98,99]. Importantly, enteric protozoa can also directly disrupt tight junctions and increase barrier permeability via the adherence of the active form of the parasite to the epithelial layer. For example, *E. histolytica* can both alter expression of and degrade zonulin-1, claudin-2, and occludin, a result of secreted cysteine protease A5 [100,101]. Similarly, *G. duodenalis* secretes cysteine proteases that can disrupt the claudin-1, claudin-4, and occludin arrangement in IECs [102,103]. Under normoxic or anaerobic conditions, *G. duodenalis* infection is associated with the disruption of epithelial junctional complexes (EJCs), which results in epithelial barrier dysfunction [104,105] (Figure 1B). *Cryptosporidium* spp. can also modulate the organization and expression of various tight junction proteins, including occludin, E-cadherin, and claudin-4; however, these changes are suggested to be mediated via the induction of protein degradation pathways rather than by the secretion of cysteine proteases by the protozoa [65]. Given the evidence of the modulation of both barrier function and HIF activation associated enteric protozoa, the role of hypoxia in modulation of IEC tight junctional complexes requires further investigation.

In the gut, protozoan infections have also been associated with microbiota dysbiosis and biofilm disruption, both of which may lead to the liberation of invasive pathobionts [106,107,108,109,110,111]. Interestingly, microbiota-derived products, including short-chain fatty acids (SCFAs) such as butyrate, play a role in the stabilization of HIF, which in turn improves epithelial barrier functions [112] (Figure 1D). In an enteroid model of hypoxia, pre-treatment or concurrent treatment with different ratios of SCFA cocktails such as acetate, propionate, and butyrate led to increased transepithelial electrical resistance, as well as increased expression of key gut barrier and metabolism genes [113]. Thus, more research is warranted to characterize changes in EJCs during protozoan infections under hypoxic conditions, as well as the role of protozoa-associated dysbiotic microbiota and derived products in the loss of barrier functions.

### 3.2. Tissue and Blood Parasites: Oxygen Metabolism and Host Hypoxic Responses

#### 3.2.1. Tissue Hypoxia

The optimal concentration of dissolved oxygen varies depending on the localization, function, and vascularization of the tissue. Although the state of physiologic hypoxia is pertinent to the gastrointestinal tract, many tissues can become hypoxic and must respond accordingly. For example, the brain must be sufficiently oxygenized (~35 mmHg) to carry out proper functions [114]. Under conditions of cerebral hypoxia due to ischemia or injury, the brain increases metabolic consumption of oxygen [114,115,116]. In the lungs, human influenza A virus infection can lead to localized hypoxia and alveolar cell death, resulting in oxygen depletion below the preferred range of 100–160 mmHg [115,117]. Alternatively, the skin functions optimally with a much lower concentration of oxygen that increases from ~8 to 35 mmHg across the superficial to the subpapillary plexus, respectively, while perturbations to these concentrations can impair keratinocyte proliferation and junction integrity [115,118]. Hence, given the various oxygenic environments sustained by each tissue, fluctuations in oxygen concentration can subject virtually any bodily tissue to hypoxia and incur damage in the absence of an appropriate cellular response.

Tissue and blood protozoan parasites are responsible for some of the most prevalent human parasitic infections, including malaria, Chagas disease, leishmaniasis, and toxoplasmosis [119]. Unlike enteric protozoa, which must be adapted to the fluctuating low levels of oxygen in the gut, tissue and blood protozoa exhibit a more diverse array of oxygenic preferences depending on where in the body they reside, and numerous investigations have aimed to characterize the hypoxic signatures of the host. Indeed, numerous tissue and blood protozoa either require or upregulate expression of HIF to enact a successful infection.

#### 3.2.2. *Leishmania* spp.

*Leishmania* spp. are a group of flagellated protozoa that are responsible for three different forms of leishmaniasis: visceral, cutaneous, and mucocutaneous [120]. The promastigote stage of the parasite replicates extracellularly within a sandfly (approximately 90 sandfly species can transmit *Leishmania*), which can then deliver the protozoan to the mammalian host during a blood meal [120,121]. In the mammalian host, obligate intracellular amastigotes will be phagocytosed by macrophages or dendritic cells, allowing them to travel to distant sites in the body [122]. A study assessing parasitic growth under anoxic conditions noted a loss of motility and increased secretion of lactate in promastigotes, illustrating their poor adaptability to anoxic conditions; hence, they are classified as aerobes [123]. Promastigotes require a high concentration of both extracellular and intracellular oxygen to produce superoxide, enabling the parasite to carry out aerobic metabolism and utilize oxygen as the final electron acceptor in the electron transport chain [124]. Additionally, all *Leishmania* spp. possess trypanothione reductase to combat oxidative stress, allowing them to survive within a macrophage [125].

**Figure 2:**
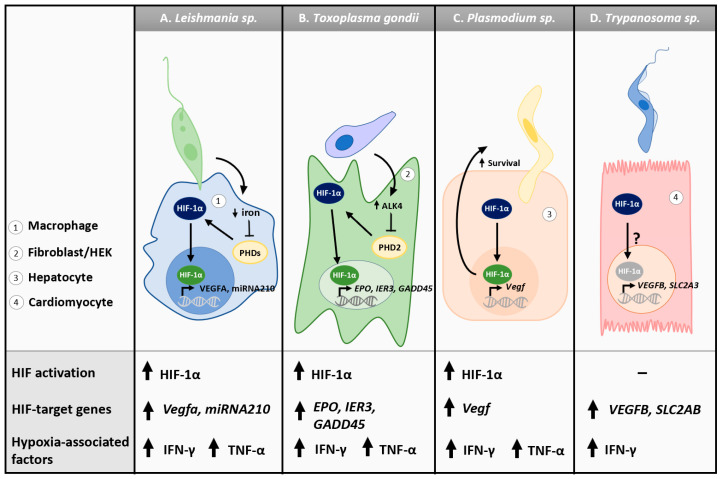
Cellular hypoxic responses to tissue and blood protozoan parasites. (**A**) *Leishmania donovani*-infected macrophages show elevated expression of HIF-1α and HIF target genes *miRNA210* and *VEGFA*. HIF-1α activation in macrophages is in part due to a decrease in intracellular iron, a cofactor of PHDs. (**B**) *Toxoplasma gondii* infection is associated with HIF-1α activation and increased expression of *IER3*, *GADD45,* and glycolytic enzymes (i.e., HK2) in fibroblasts. ALK4 phosphorylation by *T. gondii* inhibits PHD2, which further prevents the hydroxylation of HIF-1α. In HEK cells, *T. gondii* activates HIF-1α signaling to increase the production of HIF target gene products such as EPO. (**C**) *Plasmodium berghei* sporozoites activate HIF-1α and upregulate HIF target genes such as *Vegf* in murine hepatocytes. HIF-1α agonists promote *P. berghei* survival. (**D**) Hypoxia target genes *HIF-1α*, *VEGFB*, *SLC2A3*, and *ENO1*, as well as glycolytic enzymes *HK2* and *LDHA*, are upregulated in stem cell-derived cardiomyocytes infected with *Trypanosoma cruzi.* Secretion of indolepyruvate by *Trypanosoma brucei* promotes hydroxylation and subsequent degradation of HIF-1α.

Although *Leishmania* spp. are aerobic, they induce a hypoxic environment within the macrophage, increasing the expression of macrophage HIF-1α and subsequent expression of HIF-target gene micro-RNA-210 (*miRNA210*) [62,63,64,65] (Table 1). Increases in *miRNA210* enhance *L. donovani* survival as silencing of both *HIF-1*α and *miRNA210* in macrophages reduces parasitic burden and infectivity [65]. *L. donovani* also decreases the cellular iron pool, a necessary cofactor for PHDs, preventing the hydroxylation of HIF-1α [126,127] (Figure 2A). Drugs capable of targeting HIF-1α, such as resveratrol and echinomycin, decrease *L. amazonesis* survival in macrophages [128]. Recent studies have also demonstrated that HIF-dependent upregulation of *Vegfa* results in lymphangiogenesis, a critical step in the resolution of cutaneous lesions during *L. major* infection [66,129,130] (Figure 2A). The host cellular hypoxic response may also have leishmanicidal effects. Exposure of macrophages and dendritic cells to hypoxia during infection improves control of parasitic burden [131,132,133]. Mice deficient in HIF-1α in myeloid cell compartments have a more severe disease outcome and a higher parasitic burden, suggesting that HIF-1α expressed by myeloid cells contributes to the innate immune response against *L. major* [134]. Conversely, other reports suggest that HIF-1α activation has no impact on the macrophage phagocytosis of *Leishmania*, nor on control of parasitic burden [66]. HIF-1α expression is downregulated in *Leishmania-*infected hamsters in a species-dependent fashion, suggesting that different *Leishmania* spp. may elicit different hypoxic responses [135]. Finally, hyper-virulent *Leishmania* strains reduced HIF-1α expression, whereas hypo-virulent strains increased HIF-1α expression, indicating different strains within the same species can also elicit different hypoxic responses that may contribute to or mitigate virulence [136].

#### 3.2.3. *Toxoplasma gondii*

*Toxoplasma gondii* is an obligate intracellular protozoan parasite with a global prevalence between 25% and 30% [137,138]. While *T. gondii* is generally considered to be an aerobe, it must make adaptations to the environmental conditions of the definitive and intermediate hosts [137]. Indeed, *T. gondii* experiences numerous oxygenic fluctuations during its life cycle. It first colonizes the feline intestinal tract to replicate oocysts, and after sporulation and ingestion by a human, the infective tachyzoite can travel through the bloodstream to numerous bodily locations, including the central nervous system and skeletal muscles [137]. In the small intestine of felines, the merozoite form exhibits a unique gene expression profile compared to the other life stages of the parasite through the increased transcriptomic and proteomic activity of metabolic pathways, a possible adaptation to the low oxygen of the gut and increased growth requirements [139]. Interestingly, *T. gondii* encodes a prolyl-hydroxylase (TgPhyA) which hydroxylates S-phase kinase-associated protein 1, a protein that can indirectly sense oxygen levels and hence may assist with survival in various tissues [140,141]. *T. gondii* also possesses a mitochondrion that can carry out aerobic metabolism via an electron transport chain, as well as a necessary assortment of antioxidant enzymes to protect against self- and host-derived ROS [142,143,144].

In the intermediate host, *T. gondii* is associated with HIF-1α activation (Figure 2B). In murine embryonic fibroblasts, luciferase assays indicate the upregulation of HIF-1α transcription upon *T. gondii* infection [67]. Similarly, increased HIF-1α transcription is observed in human foreskin fibroblasts (HFFs) infected with *T. gondii*, which correlates with increased stable HIF-1α protein [67]. This accumulation of stable protein can be attributed to *T. gondii*’s ability to inhibit PHD2 via the stimulation of activin-like kinase 4 (ALK4) and subsequent activation of Rho GTPase and JNK MAP kinase pathways, abrogating the hydroxylation of HIF-1α [69,145,146,147]. Increasing HIF-1α production is of great importance to *T. gondii*’s fitness as it requires HIF-1α for survival and efficient replication under physiological oxygen concentrations (~3% oxygen) [67]. Moreover, *T. gondii* Cathepsin C1 can upregulate HIF-1α signaling to increase the production of EPO in HEK and HFF cells, a known HIF-dependent gene target [68]. Additionally, the expression of glycolytic enzymes is increased upon *T. gondii* infection in an HIF-dependent manner [70,148,149] (Table 1). Specifically, HK2 is delocalized from the mitochondrial membrane to the cell cytoplasm, making it more accessible to *T. gondii* [70,148,149]. The characterization of HIF activation in response to *T. gondii,* an aerobe, provides an example of a hypoxic response in normoxic conditions, suggesting that *T. gondii* may utilize host response to enhance its own fitness and virulence.

#### 3.2.4. *Plasmodium* spp.

*Plasmodium* spp. are the causative agents of malaria, a life-threatening vector-borne disease that affects nearly 250 million individuals yearly [150]. *Plasmodium* begins as a sporozoite in the insect vector, and the parasite undergoes schizogony in the liver of the infected vertebrate upon a blood meal [151]. The schizonts then become merozoites, which can infect red blood cells and subsequently digest host hemoglobin [152,153]. During the asexual stage in the human liver, sporozoites act as microaerophiles as the physiologic oxygen concentration is low [115,154,155]. Some reports suggest that sporozoite mitochondrial oxygen consumption and oxidative phosphorylation is reduced during the liver stage due to sufficient glucose availability [156,157]. However, a switch to dependence on oxidative phosphorylation is observed during the sexual life stages as it is exposed to mosquito salivary glands, which may reach up to 21% oxygen, and the capillaries of the human lung, which can approach 13% oxygen [158,159].

Numerous studies have investigated the role of HIF in *Plasmodium* spp. infections (Figure 2C). Pharmaceutical-induced activation of HIF-1α results in increased survival of *P*. *berghei* sporozoites in hepatocytes without altering the growth of the exo-erythrocytic stage of the parasite [160]. Mice infected with *P. berghei* have increased tissue levels of HIF-1α and VEGF protein, driving angiogenesis as a compensatory mechanism to increased oxygenation in hypoxic infected tissues [71]. Furthermore, insufficient expression of HIF-1α by brain cells has been suggested to contribute to the progression of cerebral malaria [161]. However, increased HIF-1α and VEGFA has not been the consensus in malaria patients. For example, while higher levels of HIF-1α mRNA expression have been identified in placental tissues, VEGFA expression was downregulated in malaria patients [162]. Additionally, post-mortem brain tissue from severe cerebral malaria patients presented no changes in HIF-1α expression but increases in VEGFA, indicating changes in VEGFA may be tissue-dependent or HIF-independent [163].

#### 3.2.5. *Trypanosoma* spp.

Chagas disease is a vector-borne parasitic infection caused by the parasite *Trypanosoma cruzi*. *T. cruzi* colonizes the gut of triatomine insects to generate infectious trypomastigotes which can then be transferred into the mammalian bloodstream during a blood meal [164]. *T. cruzi* can invade an assortment of nucleated cells but demonstrates an affinity for cardiac and skeletal muscle tissue, and therefore the amastigote is an obligate intracellular parasite [165]. From the insect vector’s gut to cardiac tissues, *T. cruzi* is exposed to diverse oxygenic states and hence exhibits variable metabolic behaviors that capitalize on fluctuating microenvironmental conditions [166]. In the low-oxygen environment of the triatomine gut, *T. cruzi* favors an anaerobic mode of metabolism by increasing glycolytic flux to sustain morphological changes [167,168,169]. In the vertebrate host’s bloodstream, trypomastigotes exhibit a preference for oxidative phosphorylation by increasing the activity of the ETC, utilizing oxygen from the blood to sustain an aerobic mode of metabolism [168,170]. *T. cruzi* can combat oxidative stress by expressing antioxidant enzymes such as peroxiredoxins and trypanothione synthetase [171]. Interestingly, it has been postulated that the presence of ROS influences the proliferation of epimastigotes, suggesting the parasite utilizes environmental oxygen cues to regulate its life cycle [172].

Few reports have characterized the hypoxic signature of *T. cruzi* infection. Leukocytes from the cardiac tissue of human patients with late-stage trypanosomiasis exhibit increased expression of HIF-1α in conjunction with increased ATP catabolism and parasitic burden [173]. Induced pluripotent stem cell-derived cardiomyocytes (iPSC-CMs) also show significant enrichment for hypoxia-related genes upon infection with *T. cruzi*, including *HIF-1α*, *VEGFB*, *HK2*, *SLC2A3*, *ENO1*, and *LDHA* [72] (Figure 1, Table 1). Importantly, reduced expression or inhibition of the glucose transporter GLUT4 in cardiomyocytes lowered parasitic uptake, suggesting *T. cruzi* can utilize GLUT4 to enter cardiomyocytes [72]. GLUT4 is a passive glucose transporter that is known to have increased action during hypoxia in an HIF-dependent manner, and *T. cruzi* may hence benefit from the hypoxic response to cellular invasion [174]. However, another report indicates that *T. cruzi* strain Tulahuen may downregulate HIF-1α in myoblasts, while no alterations to HIF-1α were observed in other strains, suggesting an isolate-dependent phenomenon [175]. Interestingly, *T. brucei*, the etiologic agent of African trypanomiasis, hydroxylates HIF-1α via the secretion of indolepyruvate, resulting in reduced macrophage glycolysis [176]. This functions as an immune evasion mechanism and prevents macrophages from shifting their metabolism to favor glycolysis, an example of an indirect way in which a parasite can mitigate hypoxic signaling [176]. This observation also supports the hypothesis that HIF-1α can play a role in host immune evasion against protozoan parasites. However, expression of HIF-1α and target genes *vegfa*, *glut1*, and *il1b* is elevated in the median eminence and hypothalamus of both Rag1^−/−^ and wild-type mice infected with *T. brucei brucei* (Tbb), suggesting different tissues and cell types may alternatively regulate HIF in the presence of the parasite [177].

## 4. Host Immune Response and Markers of Hypoxia during Protozoan Infections

Parasites elicit a broad array of host immune responses dependent on their mechanism and site of infection. Intracellular protozoans often trigger Th1 immune responses, while extracellular parasites such as *Giardia* can induce components of Th1, Th2, and Th17 immune responses [178,179]. Several cytokines can promote HIF stabilization independent of oxygen concentration, activating hypoxic responses under normoxic conditions. Proinflammatory cytokines can stabilize HIF via non-canonical pathways and can be reciprocally regulated by HIF. For instance, HIF-dependent regulation of IL-1β, TNFα, INF-γ, IL-33, IL-6, IL-12, and IL-23 illustrates the strong influence HIF has on innate immunity [180,181]. In this section, we will review the main HIF-related cytokines in the context of protozoan infections.

### 4.1. IL-1β and NLRP3

IL-1β is one of the most important modulators of both inflammation and infection. Notably, IL-1β upregulates HIF-1α protein levels in various human tissues and immune cells, which is confirmed by subsequent HIF-dependent VEGF upregulation [182,183,184,185]. This stabilization of HIF-1α occurs via the IL-1β-driven phosphorylation of extracellular signal-regulated kinase 1/2 (ERK1/2 or p42/44), activating a mitogen-activated protein kinase (MAPK) pathway responsible for subsequent HIF-1α stabilization [185,186]. Importantly, the regulation of HIF-1α by IL-1β can exhibit bidirectionality as the *IL1β* gene possesses a canonical HIF-1α binding site upstream of the transcription start site [187]. Knockout of the VHL protein in mice exacerbates IL-1β secretion, further ameliorating this relationship [188]. Accumulation of the TCA metabolite succinate has also been implicated in the HIF–IL-1β axis. During oxygenic stress, ROS can inactivate succinate dehydrogenase, halting further metabolism of succinate [189]. Macrophage-released succinate can inhibit PHDs, promoting the stabilization of HIF-1α and IL-1β signaling [187]. Alternatively, succinate can act extracellularly to promote activation of the HIF–IL-1β axis as human umbilical vein endothelial cells (HUVECs) express a surface succinate receptor (SUCNR1) which, upon recognition of macrophage-produced succinate, can upregulate IL-1β in an HIF-1-dependent manner [190]. As a potent activator of IL-1β secretion via activation of caspase-1, the nod-like receptor protein 3 (NLRP3) inflammasome also has a role in HIF-dependent gene activation [190,191]. In the gut, NLRP3 can bind the mammalian target of rapamycin (mTOR) and promote the development of intestinal inflammation during a state of hypoxia, a result that is counteracted via PHD inhibition [192]. HIF-1α also promotes NLRP3-driven microglial pyroptosis in the brain of mice with traumatic brain injuries, illustrating the tissue-independent pro-inflammatory crosstalk that exists between NLRP3 and HIF [193]. Hence, the production of cytokines can also be an indirect result of HIF-1α-promoted inflammasome assembly.

To date, no studies have demonstrated direct HIF-dependent activation of NLRP3 and the HIF–IL-1β axis by protozoa. *E. histolytica* peroxiredoxins promote IL-1β secretion and activation of the NLRP3 inflammasome via the activation of toll-like receptor 4 [194,195]. *E. histolytica* cysteine proteases can increase production of IL-1β by mimicking the action of caspase-1, activating pro-IL-1β independent of the host [196,197]. *G. duodenalis* can activate the NLRP3 inflammasome, leading to subsequent production of antimicrobial peptides [198]. As *G. duodenalis* cysteine proteases are able to cleave inflammatory cytokines and chemokines such as CXCL1, CXCL2, CXCL3, CXCL8, CCL2, and CCL20 to dampen local inflammation, this model protozoan parasite offers an interesting avenue for further research into HIF-mediated cytokines [199,200,201]. NLRP3 inflammasome activation and IL-1β secretion can also promote macrophage pyroptosis, a benefit to the parasite that may outweigh the consequential pro-inflammatory response [202,203]. Interestingly, *G. duodenalis* extracellular vesicles induce the production of IL-1β via modulation of cyclooxygenase 2 (COX2), driving macrophage secretion of the cytokine in a ROS-dependent manner via MAPK/NF-κB signaling [204,205,206]. The increased secretion of IL-1β and concomitant activation of NLRP3 has also been observed upon infection with tissue and blood parasites. For instance, *Leishmania* spp., *T. gondii*, and *T. cruzi* have been shown to activate the NLRP3 inflammasome [207,208,209,210,211,212,213,214]. Moreover, the secretion of indolepyruvate by *T. brucei* inhibits the expression of pro-IL-1β by bone marrow-derived macrophages in an HIF-1α dependent manner [176]. *Leishmania* spp. and *T. gondii* can also suppress Th1 response by secreting an IL-1β antagonist (IL1RN) [211,215,216]. While *Plasmodium spp*. infection does not cause ubiquitous increases in IL-1β, malarial hemozoin induces IL-1β secretion via activation of the NLRP3 inflammasome [217,218,219]. Further investigation into the role of the HIF–IL-1β axis during host–protozoan interactions will shed light on the physiological significance of this important interactive network.

### 4.2. TNFα

Tumor necrosis factor alpha (TNFα) is a pleiotropic pro-inflammatory cytokine which regulates inflammation and pathogenesis. TNFα promotes HIF-1 binding to DNA and elicits a hypoxic response in the absence of oxidative stress [182]. This finding is corroborated in rat peritoneal inflammatory cells, which exhibit mirrored increases in HIF-1α protein when cultured in hypoxic conditions or exposed to TNFα prior to cell injury [220]. Importantly, ROS have been identified as key mediators of hypoxic TNFα regulation. In fetal alveolar type II epithelial cells pre-treated with either antioxidants or inhibitors of the mitochondrion complex I, a pertinent element of the electron transport chain and producer of ROS, the TNFα-dependent stabilization of HIF-1α is lost [221]. Alternatively, TNFα relies on NF-κB for the activation of HIF-1α [222]. In TNFα-stimulated HEK cells treated with Iκ-b kinase (IKK) inhibitors, a kinase required for the activation of NF-κB, the transcription and translation of HIF-1α is abrogated [223,224]. IKKs can also be phosphorylated by TNFα, suggesting that TNFα activates HIF-1α expression via an IKK/NF-κB-dependent mechanism [224]. Similar results were observed in murine skeletal muscle cells as TNFα-induced NF-κB activation is required to activate HIF-1α and subsequently promote glycolytic metabolism, confirming TNFα not only activates HIF1α but also elicits a robust hypoxic response [225].

TNFα is a prominent cytokine in the immune response to several protozoan parasites (Figure 1A and Figure 2). TNFα induces a strong inflammatory state and can be cytotoxic to the host during *E. histolytica* infection, corroborated by higher TNFα serum levels in patients with more severe outcomes [226,227,228,229,230]. Interestingly, *E. histolytica* trophozoites exhibit chemotaxis towards TNFα, indicating that sites of TNFα may have increased parasitic colonization [231]. Increased production of TNFα can also be beneficial for effective parasite clearance in the context of *G. duodenalis* [86,206,232,233]. The role of TNFα in *Cryptosporidium* infections is unclear as parasite infectivity in TNFα-deficient mice is unchanged, while exogenous TNFα promotes oocyst shedding [234,235,236,237,238]. In *Leishmaniasis*, TNFα enhances disease pathogenesis, but TNFα-deficient mice can exhibit a compensatory mechanism of increased nitric oxide synthase expresssion to further perpetuate parasite killing, indicating this cytokine is not paramount for host defense [239,240]. TNFα also promotes more severe disease outcomes in the context of *Plasmodium* sp. infections as the absence of TNF receptor 2 (TNFR2) and/or administration of TNFα-neutralizing antibodies leads to an absence of cerebral malaria and fewer liver lesions [241,242,243,244]. In toxoplasmosis, TNFα knockout mice are unable to control the burden of *T. gondii* and eventually die of necrotizing toxoplasmic encephalitis, stressing the importance of this cytokine for host defense in the brain [245]. Similar findings have been illustrated in models of *T. cruzi* infection, where transgenic mice capable of neutralizing TNFα have increased infection severity and higher mortality rates compared to wild-type mice [246,247]. The mechanisms involved in the HIF-1α–TNFα interactions in the context of the host inflammatory response to protozoan parasites require further investigation.

### 4.3. INF-γ

Bolstering the relationship between hypoxia and inflammation, HIF can also be regulated by interferons (IFNs), a complex system of cytokines that orchestrate pro- and anti-inflammatory responses [248]. INF-α, INF-β, and IFN-γ can elicit an HIF-mediated response [249,250,251]. IFN-γ is a powerful immune-modulating cytokine that must be carefully calibrated to assist the host during infection and may promote varying hypoxic responses. IFN-γ increases HIF-1α activation in T84 and Caco-2 IECs when NF-κB is uninhibited [181,252]. The activity of dual oxidase II, a known generator of ROS, is also required for IFN-γ induction of HIF-1α and subsequent VEGF production in pancreatic cell lines [253]. Conversely, HIF-1α activation in Treg cells can also promote secretion of IFN-γ to potentiate Th1 immune responses [254]. Other reports suggest that IFN-γ possesses a potent ability to repress the transcription of HIF-1β, abrogating the hypoxic response in human colonic T84 cells [255]. Interestingly, IFN-γ has exhibited the ability to induce PHD3 in endothelial cells, suggesting IFN-γ may also indirectly exert HIF-1α inhibition [256].

IFN-γ contributes greatly to host protection against *E. histolytica* as higher secretion of IFN-γ inhibits parasitic ingestion and replication [257,258] (Figure 1A). In cryptosporidiosis, IFN-γ is also required for the clearance of *Cryptosporidium* [234,259] (Figure 1C). In leishmaniasis, IFN-γ is required for macrophages to facilitate nitrogen oxidation and subsequent intracellular and extracellular killing of *L. major* amastigotes [239,260] (Figure 2A). Similarly, both IFN-γ and its receptors are required for protection against *T. gondii* and *T. cruzi* [261,262,263,264,265] (Figure 2B,D). Although secretion of IFN-γ can be detrimental to the host, *Plasmodium* spp. infections do not develop into cerebral malaria in IFN-γ receptor-deficient mice or mice receiving IFN-γ-neutralizing antibodies [242,243,244,266] (Figure 2C). Much remains to be learnt about the interactive role of IFN-γ and hypoxia during protozoan infections.

### 4.4. IL-33

Interleukin-33 (IL-33) possesses an HRE in its promoter, suggesting that it can be activated by HIF [267,268]. In the gut, HIF-1α-driven expression of IL-33 can enhance inflammation in mice with dextran sulfate sodium (DSS)-induced colitis [267]. Expressed constitutively by many tissues, including blood vessels, lymphoid tissues, and epithelial cells, IL-33 acts as a ligand for IL-1 receptor-related protein ST2, found on the surface of immune cells such as mast cells and Th2 cells [269,270,271]. Additionally, IL-33 functions as an alarmin, helping mast cells recognize cellular injury and acting as one of the first responders to parasitic invasion [272,273,274]. IL-33 is inherently linked to the hypoxic response via mTOR, a serine/threonine protein kinase that regulates cell growth, proliferation, and metabolism [275,276]. The mTOR complex is expressed by numerous cell types, and mTOR complex 1 (mTORC1) specifically contributes to cellular homeostasis and modulates glucose metabolism by driving the expression of HIF-1α in a multifaceted manner [277,278,279,280]. IL-33 can activate mTOR, more specifically mTORC1, eliciting a shift favoring glycolytic metabolism and an increase in HIF-dependent VEGFA expression in various immune cells [281,282,283,284,285]. Conversely, HIF-1α can reduce ILC2 responsiveness to IL-33 and even further exacerbate IL-33 production in rheumatoid arthritis, making it a target of drug treatment and indicating that this proinflammatory-hypoxic crosstalk is bidirectional [286,287].

In mice infected with *E. histolytica*, IL-33 reliably recruits ILC2s to protect against amebic colitis but can also exacerbate amebic liver abscess pathogenesis [288,289]. Mice infected with *Cryptosporidium* exhibit elevated levels of IL-33 in the small intestine, although *C. parvum* possesses *IL33-*silencing RNA transcripts that can be transferred to the host epithelium during invasion to block the IL-33 response [290,291]. In toxoplasmosis, IL-33 plays a role in the pathogenesis of *T. gondii* in the brain, acting locally to promote astrocyte control of parasitic burden and reducing the risk of encephalitis [292,293]. Blockage of the IL-33/ST2 pathway lessens the severity of ileitis in mice induced by oral *T. gondii*, illustrating the organ-dependent response of IL-33 [294]. This contradictory role of IL-33 has also been noted during *Plasmodium* spp. infections. IL-33 prevents development of cerebral malaria (CM); however, the IL-33/ST2 pathway in mice lessens the pathogenicity of experimental cerebral malaria [295,296]. Ultimately, studies in the field have exemplified how IL-33 can either reduce the inflammatory response or exacerbate it depending on the cells it acts on and the location in the body [297]. Research is now warranted to characterize how this response interacts with hypoxia during infection.

## 5. Conclusions

In summary, HIF can be modulated by protozoa to influence the pathophysiology of parasitic infections. For tissue and blood protozoa, the role of HIF is varied and may help or hinder the parasite in establishing an infection. Little is known about the role of HIF during enteric protozoan parasitic infections, though transcriptomic studies on intestinal epithelial cells clearly point to a hypoxic signature. More research is needed to characterize how pathophysiology may be altered under hypoxic conditions during protozoan infections. Given that HIF can modulate the transcription of key pro-inflammatory mediators, the role of this transcription factor is of great importance when interrogating host immune responses against protozoa. Elucidation of the role of tissue and mucosal oxygen tension and HIF status during these parasitic infections will provide great insight regarding host–parasite interactions and may also have downstream effects on the microbial community present at the sight of infection. Moreover, microbiota dysbiosis associated with protozoan infections may impact hypoxia-related functions since bacterial-derived metabolites can directly activate HIF. Conversely, a hypoxic environment may alter local commensal communities. The effects of hypoxia on mucosal microbiota during infection and mechanisms driving homeostatic modulation will shed light on avenues with great potential for therapeutic development.

## Figures and Tables

**Table 1:** Murine and human HIF target genes upregulated upon infection with protozoan parasites.

Parasite	Glucose Metabolism Genes	Hypoxia-Related Genes	References
Enteric Parasites			
*Entamoeba histolytica*	*Pgm2*, *Gpt2*, *Pdk3*, *LDHA*, *ENO1*, *ENO2*, *PFKFB3*, *GAPDH*, *ALDOA*	*Vegfa*, *Cgref1*, *Prelid2*, *Grk3*, *Celsr3*, *Atg9b*, *Icam1*, *Gpr160*, *Jdp2*, *Ciart*, *Wdr45b*, *Slc25a36*, *Il6ra*, *Cpne8*, *Tpd52*, *Bnip3*, *Gpt2*, *Slc8a1*, *Celsr3*, *Ackr4*, *Ppan*, *Hspbap1*, *Lancl3*, *Ccng2*, *Matr3*, *Taf9b*, *Piga*, *Kcnj2*, *Cntnap1*, *Gpnmb*, *HIF1*a, *MT1G*, *MT1H*, *MT1P*, *MT2A*, *MT3*, *IER3*, *JUN*, *FOS*, *JUNB*, *JUND*, *HSP70*, *DDIT3*, *CRYAB*	[55,56]
*Giardia duodenalis*	*ENO1*, *ENO1*, *ALDOA*, *ALDOC*	*JUN*, *FOS*, *IER3*, *ANKRD37*, *GADD45A*, *IATF*, *MIR210HG*, *SLC2A3*, *NOS2*, *HILPDA*	[58,59]
*Cryptosporidium* spp.	-	*HIF1*a, *HSP70*	[60,61]
Tissue and Blood Parasites			
*Leishmania* spp.	*Hk2*, *Pfkl*, *Ldha*, *Kh3*, *Gapdh*, *Eno2*, *Pdha1*	*Hif1*a, *Hif2*a, *Vefga*, *Pik3ca*, *Plog1*, *Trf*, *Vhl*, *Plog2*, *Cul2*, *Serpine1*, *Bcl2*, *Mtor*, *Erbb2*, *Insr*, *Nos2*, *Tlr4*, *Cdkn1α*, *Hmox1*, *Akt3*, *Eif4e2*, *Il6*, *Angtp1*, *Rps6kb2*, *Egln3*, *Ifngr1*, *Mknk1*, *Eloc*, *Rps6*, *Rela*, *Egln1*, *Cdkn1b*, *Rbx1*, *Prkca*, *Camk2b*, *miRNA210*	[62,63,64,65,66]
*Toxoplasma gondii*	*HK2*, *PFK1*, *PGK1*, *ENO1*	*Hif1*a*/HIF1*a, *EPO*, *IER3*, *JUNB*, *ITF*, *ICAM1*, *BNIP3. TFR*, *GADD45*	[67,68,69,70]
*Plasmodium* sp.	-	*Vegf*, *Hif1*a	[71]
*Trypanosoma* spp.	*PFKFB3*, *PFKP*, *ENO1*, *ENO2*, *GAPDH*, *HK2*, *PGM1*, *HK1*, *LDHA*, *ALDOA*, *PKM*, *PGK1*	*HIF1*a, *VEGFB*, *NOCT*, *BHLHE40*, *GPI*, *SLC2A3*, *FOS*, *SLC2A1*, *GLRX*, *PGF*, *TPI1*, *MAFF*	[72]
